# Associations between prenatal malaria exposure, maternal antibodies at birth, and malaria susceptibility during the first year of life in Burkina Faso

**DOI:** 10.1128/iai.00268-23

**Published:** 2023-09-27

**Authors:** Hamtandi Magloire Natama, Gemma Moncunill, Marta Vidal, Toussaint Rouamba, Ruth Aguilar, Rebeca Santano, Eduard Rovira-Vallbona, Alfons Jiménez, M. Athanase Somé, Hermann Sorgho, Innocent Valéa, Maminata Coulibaly-Traoré, Ross L. Coppel, David Cavanagh, Chetan E. Chitnis, James G. Beeson, Evelina Angov, Sheetij Dutta, Benoit Gamain, Luis Izquierdo, Petra F. Mens, Henk D. F. H. Schallig, Halidou Tinto, Anna Rosanas-Urgell, Carlota Dobaño

**Affiliations:** 1 Unité de Recherche Clinique de Nanoro, Institut de Recherche en Sciences de la Santé, Direction Régionale du Centre-Ouest, Nanoro, Burkina Faso; 2 Barcelona Institute for Global Health (ISGlobal), Hospital Clínic – Universitat de Barcelona, Barcelona, Spain; 3 CIBER de Enfermedades Infecciosas (CIBERINFEC), Barcelona, Spain; 4 CIBER de Epidemiologia y Salud Pública (CIBERESP), Barcelona, Spain; 5 Infection and Immunity Program, Department of Microbiology, Monash Biomedicine Discovery Institute, Monash University, Melbourne, Victoria, Australia; 6 Centre for Immunity, Infection & Evolution, Institute of Immunology & Infection Research, Ashworth Laboratories, School of Biological Sciences, University of Edinburgh, Edinburgh, United Kingdom; 7 Malaria Parasite Biology and Vaccines Unit, Department of Parasites and Insect Vectors, Institut Pasteur, Université de Paris, Paris, France; 8 Burnet Institute, Melbourne, Victoria, Australia; 9 U.S. Military Malaria Vaccine Program, Walter Reed Army Institute of Research (WRAIR), Silver Spring, Maryland, USA; 10 Université Paris Cité, INSERM, Paris, France; 11 Academic Medical Centre at the University of Amsterdam, Amsterdam, the Netherlands; 12 Department of Biomedical Sciences, Institute of Tropical Medicine, Antwerp, Belgium; University of California Davis, Davis, California, USA

**Keywords:** malaria, *Plasmodium falciparum*, pregnancy, prenatal exposure, maternal antibodies, cord blood, protection, childhood

## Abstract

In this study, we investigated how different categories of prenatal malaria exposure (PME) influence levels of maternal antibodies in cord blood samples and the subsequent risk of malaria in early childhood in a birth cohort study (*N* = 661) nested within the COSMIC clinical trial (NCT01941264) in Burkina Faso. *Plasmodium falciparum* infections during pregnancy and infants’ clinical malaria episodes detected during the first year of life were recorded. The levels of maternal IgG and IgG_1-4_ to 15 *P*. *falciparum* antigens were measured in cord blood by quantitative suspension array technology. Results showed a significant variation in the magnitude of maternal antibody levels in cord blood, depending on the PME category, with past placental malaria (PM) more frequently associated with significant increases of IgG and/or subclass levels across three groups of antigens defined as pre-erythrocytic, erythrocytic, and markers of PM, as compared to those from the cord of non-exposed control infants. High levels of antibodies to certain erythrocytic antigens (i.e., IgG to EBA140 and EBA175, IgG1 to EBA175 and MSP1_42_, and IgG3 to EBA140 and MSP5) were independent predictors of protection from clinical malaria during the first year of life. By contrast, high levels of IgG, IgG1, and IgG2 to the VAR2CSA DBL1-2 and IgG4 to DBL3-4 were significantly associated with an increased risk of clinical malaria. These findings indicate that PME categories have different effects on the levels of maternal-derived antibodies to malaria antigens in children at birth, and this might drive heterogeneity to clinical malaria susceptibility in early childhood.

## INTRODUCTION

Infants are thought to be protected against malaria during the first months of life mainly due to the transplacental passage of maternal antibodies as well as additional factors such as fetal hemoglobin and behavioral practices reducing exposure. However, in high-transmission settings, malaria in early infancy is common, and susceptibility to the infection varies between individuals. Indeed, birth cohort studies across different African countries showed that up to 59% of infants can experience at least one clinical episode of malaria during the first year of life in certain transmission settings (1 – 4). In consequence, despite the potential protective effect of maternally transferred antibodies to newborns, infants living in high-transmission areas may be highly affected by malaria infections and disease, raising controversy on the role of maternal antibodies in malaria protection during the first months of life (5).

Early in life, the immune system relies mostly on innate immunity and maternal antibodies acquired *in utero* and through breastfeeding (6 – 9). Immunoglobulin G (IgG) is the only antibody isotype that can cross the placenta and it reflects the immunological experience of the mother in her living environment (10, 11). In malaria endemic settings, antibodies against diverse *Plasmodium falciparum* antigens have been detected in cord and/or peripheral blood of the newborn at birth (12 – 15).

The protection provided by maternal antibodies against malaria in infants has been evidenced by several studies that showed associations between maternally transferred antibodies and different malaria-related indicators. Early observations from epidemiological studies suggested that infants in endemic areas are relatively protected from clinical episodes and/or severe malaria. However, such protection (i.e., from clinical cases) was demonstrated only in few studies (16 – 18). Maternal antimalarial antibodies have also been associated with protection from malaria infection (19) and with a delayed time-to-first parasitemia in early childhood (20, 21). Other studies have shown the ability of some infants in endemic settings to maintain parasite density at a very low levels for weeks or even months without developing clinical symptoms (22 – 24). Those low-density infections are frequently spontaneously cleared, suggesting a protective effect of maternal antibodies (24 – 26). However, other studies failed to find any association between maternal antibodies and malaria in early infancy (1, 27 – 29) and, in some cases, even an increased risk has been reported (27, 30, 31). In such context, factors that modulate malaria risk/protection in early childhood are not fully understood and further field studies addressing the role of maternal antibodies are especially needed.

Variation of malaria susceptibility at individual level could be partially explained by a differential protective effect of maternal antibodies as their levels may vary from one newborn to another (15, 16, 20). We have previously reported that *in utero* exposure to malaria parasites and/or antigens have a profound effect on fetal innate immunity, which affects malaria risk during the first year of life (32). In this study, we examined how prenatal malaria exposure (PME) (and other covariates) could influence the levels of maternal antibodies in cord blood at birth and the impact of maternal antibody concentrations on subsequent risk of malaria in early childhood. We hypothesized that placental malaria (PM) exposure (PME) drives the heterogeneity of maternal antibody levels at birth and, depending on the time and type of malaria exposure during pregnancy, levels of maternal antibodies and subsequent protective effect against malaria may vary among children. Depicting the effect of PME on maternally transferred antibodies and its consequences on infant’s health is of high importance because despite the adoption of malaria in pregnancy preventive treatment with sulfadoxine-pyrimethamine (IPTp-SP) by most of sub-Saharan African countries, as recommended by WHO, children are often born from mothers with peripheral and/or PM (33 – 35).

## MATERIALS AND METHODS

### Study design and participants

A prospective birth cohort study was nested within the COSMIC trial (NCT01941264). In brief, COSMIC was a cluster-randomized controlled trial investigating the protective efficacy of adding community-scheduled screening and treatment (CSST) of malaria during pregnancy to the standard IPTp-SP (CSST/IPTp-SP, intervention arm) compared to IPTp-SP alone (control arm) in Burkina Faso, Benin, and The Gambia (36). The CSST extension strategy was implemented through monthly screening and treatment of malaria infection with artemether-lumefantrine (AL) by community health workers using rapid diagnostic tests (RDTs). In both arms, a health assessment was carried out at each antenatal care (ANC) visit until the time of delivery. Suspected malaria cases had a RDT and women testing positive were treated with AL. For each RDT performed, a blood sample was collected for a blood smear and dried blood spots (DBS) on filter paper for malaria diagnosis by light microscopy and polymerase chain reaction (PCR), respectively. At the time of delivery, peripheral and cord blood samples were collected for blood smears and DBS for later parasitological diagnosis, and a placenta biopsy for histology. Current health status and birth outcomes were collected. All newborns were physically examined and weighed on digital scales immediately after delivery. Gestational age was estimated using the Ballard score.

In Burkina Faso, from the 1,800 pregnant women participating in the COSMIC trial, 734 mother-child pairs were enrolled in a birth cohort study with a 12-month follow-up. Of the 734 mother-child pairs, 661 mothers and their offspring were included for the present study. Mother-child pairs not included were those for whom complete data on history of malaria infection during pregnancy and/or cord blood samples for immunological assays were not available. The study was conducted in the health district of Nanoro, a rural area located in Centre-West of Burkina Faso (37). Malaria transmission in the region is seasonal and hyperendemic with the highest transmission period lasting from July to December and overlapping with the rainy season. The dry season lasts from January to June and corresponds to the low transmission season.

### Recruitment and follow-up

The recruitment and the follow-up procedures of the birth cohort study have been described elsewhere (3). Briefly, pregnant women from Nanoro participating in the COSMIC trial were asked at antenatal care visits to participate in the birth cohort study prior to delivery. At delivery, healthy newborns with their mothers were enrolled after informed consent was obtained. Clinical malaria episodes in infants were monitored by passive case detection, for which mothers were encouraged to seek care at peripheral health centers of their village at any time the parents or caregivers felt the infant was unwell. At each attendance to health facilities, a clinical examination of the infant was performed, and mothers were asked for previous health events. In the case of fever (axillary temperature ≥ 37.5°C) or history of fever in the previous 24 h, a malaria RDT was performed, and positive infants were treated according to national guidelines. Infants were followed up from birth to 12 months of age. The study was approved by the institutional ethics committees of Centre Muraz in Burkina Faso (006–2014/CE-CM), the Institute of Tropical Medicine in Belgium (953/14), and the University Hospital in Antwerp (UZA) in Belgium (14/26/277). Written informed consent was obtained from all mothers.

### Sample collection

Procedures have been described elsewhere (38). In brief, approximately 200 µL of maternal peripheral blood was obtained by finger-prick for blood smear preparation and blood spot on filter paper at delivery. A placental tissue section was collected from the maternal side and preserved in 10% neutral buffer formalin at 4°C for histology examination. In addition, cord blood (approximately 10 mL) was collected in heparin containing tubes by venipuncture of the umbilical vein and transferred from the peripheral health centers to the laboratory at the Clinical Research Unit of Nanoro (CRUN) for processing within 4 h. Part of the cord blood sample was used to prepare thick blood smears for LM examination and DBS on filter paper for posterior *P. falciparum* diagnosis by qPCR. Cord blood plasma samples were collected following a centrifugation at 3,000 rpm for 10 min, then frozen at −80°C. The plasma samples were subsequently shipped frozen to ISGlobal (Barcelona, Spain) for antibody quantification. For after birth malaria surveillance, peripheral blood was collected by finger-prick from each infant visiting the health facilities with presence of fever or history of fever in the previous 24 h, and used for RDT, blood smear, and spots on filter paper (Whatman 3 MM).

### Antibody assays

Quantitative suspension array technology (qSAT) applying the xMAP technology (Luminex Corp., Texas) was used to measure antibody levels to 15 *P*. *falciparum* 3D7/NF54 strain antigens. These included (i) pre-erythrocytic sporozoite antigens [i.e., circumsporozoite full length protein (CSP-fl), C-terminal end of CSP (CSP-Ct), and the NANP repeat central region of CSP (CSP-NANP)] (39); (ii) blood-stage antigens [i.e., apical membrane antigen-1 (AMA1) (40), C-terminal 42 kDa cleavage of merozoite surface protein 1 (MSP1_42_) (41), merozoite surface protein 2 (MSP2) (42), merozoite surface protein 3 (MSP3) (43), merozoite surface protein 5 (MSP5) (44), erythrocyte binding antigen (EBA) 140 (EBA140) (45), EBA175 (46), glutamic acid rich protein (GARP) (47), reticulocyte-binding protein homolog-5 (Rh5) (48)]; and (iii) markers of placental malaria [i.e., Duffy Binding like (DBL) domains 1 and 2 (DBL1-2) and the domains 3 and 4 (DBL3-4) of, respectively, the 3D7 and FCR3 VAR2CSA variants of the erythrocyte membrane protein 1 (PfEMP1)] (49, 50). A glycan α-galactose (αGal) antigen was also included for its previous association with risk/protection of malaria during the first months of life (51). The selection of these antigens was done based on prominent targets of immunity, vaccine candidates, or prior association with protection in seroepidemiological studies or animal models (40 – 46, 48, 52, 53). Test samples were assayed singly at one dilution for IgG (1/250) and IgG_1-4_ subclasses (1/100). For all assays, 12 serial dilutions 1:2 of a positive control were used to perform antigen-isotype/subclass specific standard curves. The positive control consisted of a WHO Reference Reagent for anti-malaria *P. falciparum* human serum (NIBSC code: 10/198) at 1:50. A total of 10 different samples from malaria-naïve adult donors were used as negative controls. Two blanks were added to each plate for quality control purposes. In brief, antigen-coupled beads were added to a 384-well μClear flat bottom plate (Greiner Bio-One) in multiplex (2,300 microspheres/analyte/well) resuspended in 90 µL of luminex buffer (PBS, 1% BSA, 0.05% Azide pH 7.4). Ten microliters of sample, negative or positive controls, and blanks were added to multiplex wells and incubated during 1 h at room temperature in a shaker at 900 rpm and protected from light. Plates were washed three times with 200 µL/well of wash buffer (PBS-Tween 20 0.05%) using an automatic plate washer machine (Biotech 405TS). Then, for IgG, 25 µL of goat anti-human IgG-phycoerythrin (PE) (GTIG-001, Moss Bio) were added diluted in luminex buffer (1/400). For IgG1 and IgG3 subclasses, 25 µL of biotinylated secondary antibody (ab99775, Abcam and B3523, Merck, respectively) were added diluted in luminex buffer (1/2,000 and 1/250, respectively). For IgG2 and IgG4 subclasses, 25 µL of mouse anti-human IgG2 and IgG4 (MA1-34755 and MA5-16716, Life technologies) were added diluted in luminex buffer (1/250 both), followed by 25 µL of biotinylated anti-mouse IgG (B7401) diluted in luminex buffer (1/2,500). All antibody incubations were performed for 30 min, at room temperature, at 900 rpm, and protected from light. Next, 25 µL of streptavidin-R-phycoerythrin (42250, Merk) diluted 1/500 in Luminex buffer were added to all wells for all the antibodies and incubated 30 min, at room temperature, 900 rpm, and protected from light. Plates were washed as before and resuspended in 80 µL/well of Luminex buffer. Plates were read using a FLEXMAP 3D analyzer and at least 50 microspheres per analyte were acquired per sample. Data were captured using xPonent software. Antibody levels were reported as median fluorescence intensity (MFI).

### Malaria detection and definitions

SD-Bioline malaria antigen P.f test (05FK50, Standard Diagnostics, Inc, Korea) detecting PfHRP2 was used for malaria RDT according to the manufacturer’s instructions. The microscopic examination of thick blood smears stained with Giemsa (10%) was performed according to standard procedures (54). DBS on filter paper (from peripheral and cord blood) were used for DNA extraction (QIAamp 96 DNA blood kit, Qiagen, Germany) and *P. falciparum* detection of *Pf*-varATS by qPCR, as previously described (38). Data on past history of malaria infections during pregnancy and histological examination of placental tissues were obtained from the COSMIC trial (36). A clinical malaria episode during the first year of life was defined as the detection of *P. falciparum* parasites by qPCR in peripheral blood of children and presence of fever or history of fever in the previous 24 h. PM infections were categorized by histological examination as follows: (i) acute infection (parasites present, malaria pigment absent), (ii) chronic infection (parasites and malaria pigment present), (iii) past infection (parasites absent but pigment present), and (iv) no infection (both parasites and malaria pigment absent). PME was categorized based on placental infection (past, chronic, acute) and maternal peripheral infection either during monthly screening, ANC visits, unscheduled visits or at delivery. The “exposure No PM’’ status was defined as presence of a documented peripheral infection during pregnancy but with no evidence of PM. Congenital malaria infection was defined as the presence of *P. falciparum* parasites in the cord blood as detected by qPCR. Non-exposure of newborns to malaria during pregnancy was defined as absence of documented peripheral and placental malaria infection during the course of pregnancy and at delivery.

### Statistical analysis

MFI measurements were transformed to log_10_ scale for the subsequent analyses. The assay quality control for each antigen and plate was based on visual inspection of the performance of the standard curves and of boxplots [with medians and interquartile ranges (IQR)] of the different sample types (i.e., test samples, positive controls, negative controls and blanks).

Analyses included either all subject sample or samples stratified by PME categories and other maternal and infant covariates including malaria in pregnancy preventive treatment strategy (MiP strategy), gravidity, insecticide treated net (ITN) use by the mother, prematurity (gestational age at delivery between 28 and 36 weeks as estimated by Ballard score), low birth weight (LBW, birth weight <2,500 g), congenital malaria, infant sex, ethnicity, and transmission intensity at birth [i.e., high-transmission season (July to December) versus low transmission season (January to June)]. Comparisons of crude levels of IgG and subclasses (log_10_ MFI) across antigens and between study groups were done through boxplots with medians and IQR, by Wilcoxon rank-sum test and *P*-values adjusted for multiple comparisons all together for IgG and subclasses by Benjamini-Hochberg correction. Radar plots were also used to visualize IgG and subclasses profiles across the different PME categories. Associations between antibodies were explored by Spearman correlations across antibodies levels (in a heatmap, together with scatterplots by antigens).

Linear regression models were used to evaluate the effect of different types of PME on antibody levels at birth adjusting by confounding factors. First univariable models were fitted followed by multivariable linear regression models [coefficient, 95% confident interval (CI), *P*-values]. The associations between potential confounding/interaction factors among maternal/infants covariates and antibody levels were explored, and those factors associated were used to adjust multivariable analyses when assessing the effect of PME on antibody levels.

The associations between antibody levels (as continuous variables) at birth and the risk of clinical malaria during the first year of life were assessed in univariable and multivariable Cox proportional-hazard models. Proportionality of hazards assumption and functional form of each variable adjusted in the Cox models were tested using Schoenfeld residuals analysis and p-splines functions, respectively. Secondary variables (maternal and infant covariates) that showed significant associations with malaria during the first 12 months of life were determined in Kaplan-Meier survival analyses (log-rank test *P*-value <0.05) and included in the Cox proportional-hazard regression models (i.e., PME, LBW, birth season, and MiP strategy). *P*-value <0.05 was considered statistically significant. Ratios of protective antibodies relative to antibodies associated with increased risk of clinical malaria (the markers of PM) from the Cox proportional-hazard analyses were used to further assess the effect of maternal antibodies on malaria susceptibility during the first year of life. Values of the ratios above 1 were considered as high levels of protective antibodies as compared to that of the antibodies associated with PM. All analyses were performed using R statistical package version 3.2.3 (55).

## RESULTS

### Characteristics of study participants

The characteristics of study participants for the overall cohort and by PME categories are presented in Table 1. The mean age of pregnant women at enrollment was 26.4 years and most of them were multigravida (66.4%). The majority of deliveries (60%) occurred during the malaria high-transmission season (July–December). The mean birth weight of the newborns was 3,019 g, while 8.8% had an LBW and preterm birth occurred among 4.2% of the study participants. In total, 498 newborns (75.3%) were exposed to malaria parasites and/or antigens during pregnancy. Half of the newborns were born to mothers with past PM (51%) while 7% of them were born to mothers with chronic PM and 1.5% to mothers with acute PM at delivery. There was a good balance between males and females (48.9% and 51.1%, respectively) and the large majority of children belonged to the Mossi ethnical group (90.5%).

**TABLE 1:** Characteristics of study participants^*b*^

Characteristics	Overall cohort (*N* = 661)	Non-exposed [*N* = 163 (24.7%)]	Exposed no PM [*N* = 105 (15.9%)]	Past PM [*N* = 337 (51.0%)]	Chronic PM [*N* = 46 (6.9%)]	Acute PM [*N* = 10 (1.5%)]	*P*-value
**Maternal characteristics**							
Age (years, mean ± SD)	26.4 ± 6.24	28.2 ± 6.11	27.8 ± 6.6	25.9 ± 6.1	23.9 ± 5.7	28.8 ± 6.2	<0.001
Gravidity [*N* (%)]	–	–	–	–	–	–	<0.001
Primigravida	116 (17.5)	26 (15.9)	8 (7.6)	65 (19.3)	17 (37.0)	0 (0.0)	–
Secundigravida	106 (16.0)	19 (11.6)	14 (13.3)	64 (19.0)	8 (17.4)	1 (10.0)	–
Multigravida	439 (66.4)	118 (72.4)	83 (79.0)	208 (61.7)	21 (45.7)	9 (90.0)	–
MiP strategy in COSMIC trial [*N* (%)]	–	–	–	–	–	–	0.539
Standard IPTp-SP	326 (49.3)	93 (57.0)	50 (47.6)	157 (46.6)	19 (41.3)	7 (70.0)	–
CSST/IPTp-SP	335 (50.7)	70 (43.0)	55 (52.4)	180 (53.4)	27 (58.7)	3 (30.0)	–
Gestational age at enrollment (mean ± SD)	22.9 ± 5.9	22.7 ± 7.2	23.1 ± 5.8	23.3 ± 5.0	22.6 ± 4.2	21.9 ± 6.4	0.739
ITN use [*N* (%)]	515 (77.9)	145 (88.9)	85 (81.0)	253 (75.1)	26 (56.5)	6 (60.0)	<0.001
**Infant characteristics**							
Gender [males, *N* (%)]	323 (48.9)	84 (51.5)	52 (49.5)	159 (47.2)	23 (50.0)	5 (50.0)	0.991
Birth season [malaria high-transmission season, *N* (%)]	396 (59.9)	80 (40.1)	62 (59.0)	200 (59.3)	44 (95.7)	10 (100.0)	<0.001
Preterm birth^*a*^	28 (4.2)	6 (3.7)	2 (1.9)	15 (4.4)	5 (8.7)	0 (0.0)	<0.001
Birth weight (g, mean ± SD)	3019.0 (431.4)	2970.6 ± 432.4	3082.3 ± 402.1	3018.7 ± 432.6	2962.7 ± 483.7	3019.5 ± 324.9	0.356
LBW (<2,500 g) [*N* (%)]	58 (8.8)	17 (10.4)	5 (4.8)	29 (8.6)	6 (13.0)	1 (10.0)	0.467
Ethnicity	–	–	–	–	–	–	0.006
Mossi	598 (90.5)	152 (93.2)	96 (91.4)	304 (90.2)	39 (84.8)	7 (70.0)	–
Gourounsi	57 (8.6)	7 (4.3)	7 (6.7)	33 (9.8)	7 (15.2)	3 (30.0)	–
Fulani	5 (0.8)	3 (2.8)	2 (1.9)	0 (0.0)	0 (0.0)	0 (0.0)	–
Samo	1 (0.2)	1 (0.9)	0 (0.0)	0 (0.0)	0 (0.0)	0 (0.0)	–
Follow-up time (mean months ± SD)	11.4 ± 2.00	11.5 ± 1.8	11.6 ± 1.6	11.6 ± 2.0	11.1 ± 2.2	11.6 ± 1.0	0.728
Clinical malaria episode [*N* (%)]	402 (60.8)	87 (53.4)	67 (63.8)	213 (63.2)	30 (65.2)	5 (50.0)	0.232
Time to first clinical malaria episode (median of months)	9.9	9.6	9.0	9.8	10.5	11.5	0.047

^*a*^ Preterm birth defined as gestational age at delivery between 28 and 36 weeks as determined by Ballard score.

^*b*^ PM: placental malaria; SD: standard deviation; LBW: low birth weight; ITN: insecticide-treated net; MiP: malaria in pregnancy; COSMIC: community-based scheduled screening and treatment of malaria in pregnancy, a cluster randomized trial; IPTp-SP: intermittent preventive treatment during pregnancy with sulfadoxine-pyrimethamine; CSST/IPTp-SP: community-based scheduled screening and treatment of malaria in combination with the standard IPTp- SP.

### *P. falciparum* specific maternal antibodies profile in the cord blood

**Fig 1:**
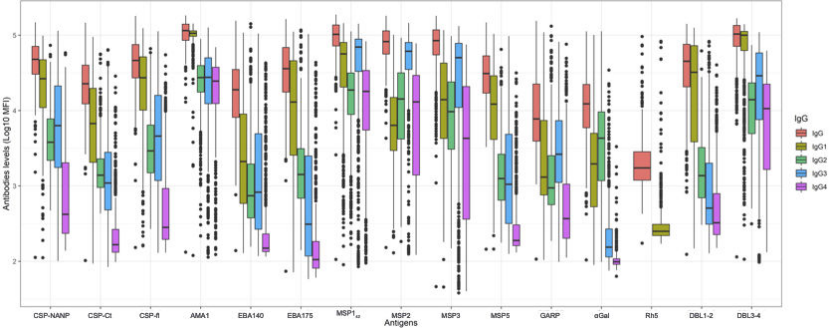
Maternal antibody profiles in cord blood against the selected antigens. Boxplots are shown with median and interquartile ranges for IgG (orange), IgG1 (olive green), IgG2 (green), IgG3 (blue), and IgG4 (purple). Antibody levels to Rh5 are only shown for IgG and IgG1 since the assay was not validated for IgG2, IgG3, and IgG4.

Figure 1 shows the profile of maternal antibodies against the selected antigens in cord blood at delivery. Maternal antibodies were detected in cord blood at different levels depending on the antigen and the IgG subclass. IgG1 and IgG3 were the predominant subclasses followed by IgG2 and IgG4. Levels of IgG and IgG subclasses against blood stage antigens (e.g., AMA1, MSP1_42_, MSP2, and MSP3) and markers of PM (e.g., DBL3-4) were higher compared to other antibodies. The Spearman correlation matrix showed a positive moderate correlation between antigens for IgG and all the subclasses (Fig. S1). The strongest correlations were observed between the CSP antigens among them (i.e., CSP-fl, CSP-NANP, and CSP-Ct) and between the DBL domains among them (i.e., DBL1-2 and DBL3-4). There was no strong correlation between antibodies to the different groups of antigens.

### Association between PME and maternal antibody levels at birth

**Fig 2:**
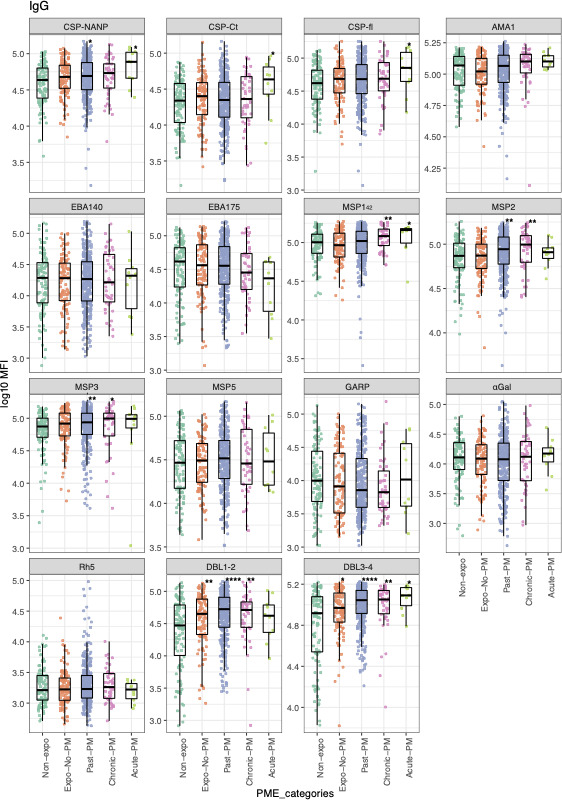
Maternal IgG levels in cord blood at birth according to PME categories. Boxplots comparing total IgG levels as log_10_ of median fluorescence intensity (MFI) between the different PME groups: Non-expo, non-exposed (in green); Expo-no-PM, Exposed/no placental malaria (in orange); Past-PM, past placental malaria (in blue); Chronic-PM, chronic placental malaria (in pink); Acute-PM, acute placental malaria (in olive green). *P*-values were determined by Wilcoxon rank-sum test using the non-exposed group as reference: *≤0.05, **≤0.01, ***≤0.001, and ****≤0.0001. *P*-values were adjusted for multiple comparisons by Benjamini-Hochberg correction.

Variation of maternal antibody levels by PME category is shown using boxplots (Fig. 2; Fig. S2) and radar plots (Fig. S3). The results show that each PME category associates to different maternal antibody profiles in the newborns as compared to those in the non-exposed control group. Differences were observed for several antigens from different parasite stages, with past and chronic PM more frequently associated with statistically significant increase of IgG and/or subclass levels against CSP (consistently for CSP-Ct, CSP-NANP, and CSP-fl) and blood-stage antigens (EBA140, EBA175, MSP1_42_, MSP2, MSP3, MSP5, GARP, Rh5). In addition, acute PM and exposed/No PM cases were, respectively, associated with increased levels of IgG1 and IgG3 against pre-erythrocytic antigens. All categories of PME were associated with significantly higher levels of IgG and/or subclasses against the markers of PM (i.e., DBL1-2 and DBL3-4) compared with that among the non-exposed control group (ANOVA, *P* < 0.05). However, past and chronic PM were more frequently associated with higher IgG levels against DBL1-2 and DBL3-4 than the other categories of PME (e.g., acute PM and exposed-no PM).

Differences in maternal antibodies by PME were further investigated using multivariable linear regression models adjusting for potential confounders. The co-factors affecting antibody levels for each antigen and included in subsequent models are listed in Table S1. Results confirmed that infants born to mothers with past PM had significantly higher levels of IgG against CSP-NANP compared to non-exposed individuals (Table 2). Similarly, past PM was associated with higher levels of IgG3 against CSP-Ct, CSP-NANP, and CSP-fl. Levels of cytophilic antibodies (i.e., IgG1 and IgG3) against CSP antigens were also significantly higher among infants born to mothers with acute PM (e.g., IgG1 against CSP-Ct and CSP-NANP) and to mothers exposed to malaria during pregnancy but with no evidence of PM at delivery (e.g., IgG3 against CSP-Ct, CSP-NANP, and CSP-fl). Levels of non-cytophilic antibodies (i.e., IgG2 and IgG4) against CSP antigens were not affected by PME categories except for IgG2 against CSP-NANP among children born to mothers with chronic PM (Table 2). For blood-stage antigens, past PM was associated with higher levels of IgG1 against Rh5 and IgG2 against MSP2, whereas chronic PM was associated with higher levels of IgG3 against EBA175 and IgG4 against MSP1_42_ as compared to those in the non-exposed control group. By contrast, chronic and past PM were associated with significantly lower levels of IgG subclasses against some erythrocytic antigens (IgG1 against MSP3 and IgG2 against MSP5 and GARP) (Table 2). The multivariable linear regression models also confirmed that all PME categories were significantly associated with increased antibody levels (IgG and subclasses) against the two markers of PM as compared to those in the non-exposed control group (Table 2).

**TABLE 2:** Multivariable linear regression models assessing the effects of the different prenatal malaria exposure (PME) categories on maternal antibodies in cord blood at birth^*a*^

Antibody-Antigen^*b*^ pairs		Exposed/no PM vs non-exposed		Past PM vs non-exposed		Chronic PM vs non-exposed		Acute PM vs non-exposed	
		Coeff (SE)	*P*	Coeff (SE)	*P*	Coeff (SE)	*P*	Coeff (SE)	*P*
**IgG**									
Pre-erythrocytic	CSP-NANP	0.05 (0.04)	0.194	**0.06** (**0.03**)	**0.040**	0.04 (0.05)	0.367	0.16 (0.08)	0.060
Erythrocytic	EBA175	0.01 (0.05)	0.870	0.02 (0.04)	0.651	0.05 (0.07)	0.509	**0.28 (0.13)**	**0.038**
Markers of PM	DBL1-2	**0.19 (0.05)**	**<0.001**	**0.34 (0.04)**	**<0.001**	**0.38 (0.07)**	**<0.001**	**0.14 (0.13)**	0.274
	DBL3-4	**0.13 (0.03)**	**<0.001**	**0.23 (0.02)**	**<0.001**	**0.25 (0.04)**	**<0.001**	**0.20 (0.07)**	**0.004**
**IgG1**									
Pre-erythrocytic	CSP-Ct	0.07 (0.08)	0.409	0.05 (0.07)	0.470	0.05 (0.11)	0.654	**0.46**(**0.21**)	**0.025**
	CSP-NANP	0.08 (0.06)	0.178	0.06 (0.05)	0.196	0.06 (0.08)	0.497	**0.29** (**0.15**)	**0.049**
Erythrocytic	MSP1_42_	**0.16** (**0.08**)	**0.045**	0.09 (0.06)	0.161	0.06 (0.10)	0.537	0.02 (0.19)	0.898
	MSP3	0.03 (0.07)	0.718	0.05 (0.06)	0.462	**0.24** (**0.07**)	**0.043**	0.10 (0.12)	0.630
	Rh5	0.01 (0.04)	0.756	**0.07** (**0.03**)	**0.043**	0.02 (0.05)	0.709	0.01 (0.10)	0.905
Markers of PM	DBL1-2	**0.36** (**0.09**)	**<0.001**	**0.60** (**0.08**)	**<0.001**	**0.78** (**0.12**)	**<0.001**	0.35 (0.22)	0.124
	DBL3-4	**0.31** (**0.06**)	**<0.001**	**0.47** (**0.05**)	**<0.001**	**0.55** (**0.07**)	**<0.001**	**0.40** (**0.14**)	**0.004**
**IgG2**									
Pre-erythrocytic	CSP-NANP	0.09 (0.05)	0.073	0.06 (0.04)	0.127	**0.15** (**0.07**)	**0.036**	0.16 (0.12)	0.215
Erythrocytic	MSP2	0.12 (0.07)	0.108	**0.15** (**0.06**)	**0.013**	0.17 (0.10)	0.082	0.19 (0.18)	0.301
	MSP5	0.06 (0.06)	0.257	**0.12** (**0.05**)	**0.013**	0.12 (0.08)	0.120	0.05 (0.12)	0.705
	GARP	0.05 (0.07)	0.523	**0.15** (**0.06**)	**0.009**	0.10 (0.09)	0.284	0.15 (0.17)	0.378
Markers of PM	DBL1-2	0.11 (0.06)	0.053	**0.16** (**0.05**)	**<0.001**	**0.19** (**0.07**)	**0.011**	0.05 (0.13)	0.695
	DBL3-4	**0.19** (**0.24**)	**<0.001**	**0.30** (**0.22**)	**<0.001**	**0.46** (**0.26**)	**<0.001**	**0.15** (**0.42**)	**<0.001**
**IgG3**									
Pre-erythrocytic	CSP-fl	**0.20** (**0.09**)	**0.029**	**0.20** (**0.09**)	**0.024**	0.18 (0.08)	0.161	0.26 (0.22)	0.243
	CSP-Ct	**0.23** (**0.08**)	**0.004**	**0.17** (**0.06**)	**0.011**	0.15 (0.10)	0.155	0.31 (0.18)	0.106
	CSP-NANP	**0.23** (**0.07**)	**0.013**	**0.13** (**0.07**)	**0.048**	0.15 (0.07)	0.241	0.25 (0.12)	0.273
Erythrocytic	EBA175	0.04 (0.12)	0.731	0.05 (0.09)	0.591	**0.35** (**0.15**)	**0.023**	0.34 (0.28)	0.228
Markers of PM	DBL1-2	**0.21** (**0.08**)	**0.010**	**0.23** (**0.07**)	**<0.001**	**0.29** (**0.11**)	**0.008**	0.08 (0.20)	0.703
	DBL3-4	**0.23** (**0.08**)	**0.006**	**0.37** (**0.07**)	**<0.001**	**0.52** (**0.11**)	**<0.001**	0.32 (0.21)	0.124
**IgG4**									
Erythrocytic	MSP1_42_	0.05 (0.09)	0.625	0.06 (0.07)	0.465	**0.25** (**0.12**)	**0.044**	0.25 (0.28)	0.273
Markers of PM	DBL1-2	0.08 (0.06)	0.204	**0.21** (**0.05**)	**<0.001**	**0.21** (**0.09**)	**0.017**	0.18 (0.16)	0.248
	DBL3-4	**0.26** (**0.08**)	**0.001**	**0.50** (**0.06**)	**<0.001**	**0.65** (**0.11**)	**<0.001**	0.20 (0.19)	0.307

^*a*^ PM, placental malaria; Coeff, coefficient; SE, standard error; *P*, *P*-value.

^*b*^ Only antibodies to antigens for which the concentrations are significantly modified by PME categories (i.e., Exposed/No PM, Past PM, Chronic PM, and Acute PM) are presented. Non-exposed category defined as absence of documented peripheral and PM infections during the course of pregnancy and at delivery was used as reference in each model. The coefficients with SE and *P*-values for PME categories significantly associated to IgG and subclasses levels are shown in bold. These models have been adjusted by confounding variables indicated in Table S1.

### Association between antibody levels at birth and risk of clinical malaria during the first year of life

**Fig 3:**
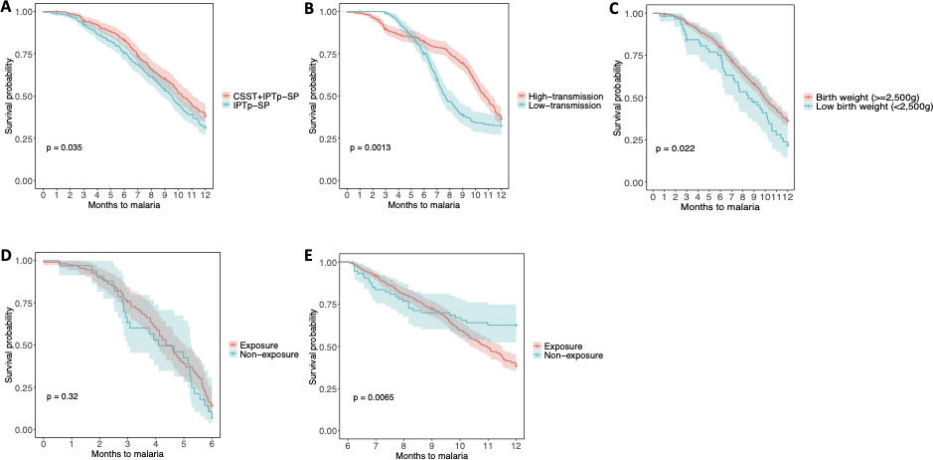
Kaplan-Meier survival curves showing the risk of clinical malaria during the first year of life for those maternal and infant’s covariates significant associated. (**A)** Risk of clinical malaria during the first year of life by malaria in pregnancy preventive treatment with Kaplan-Meier survival curves stratified by infants born to mothers who received the CSST + IPTp SP strategy (Community-based scheduled and treatment of malaria during pregnancy in addition to the standard IPTp-SP, red line) and the standard IPTp-SP alone (blue line). (**B)** Risk of clinical malaria during the first year of life by birth season stratified by infants born during malaria high-transmission season (July–December, red line) and low-transmission season (January–June, blue line). (**C)** Risk of clinical malaria during the first year of life by birth weight stratified by infants born with a birth weight ≥2,500 g (red line) and with a birth weight <2,500 g (blue line). (D and E) Risk of clinical malaria during the first year of life, by prenatal malaria exposure (any category of exposure vs non-exposure) during the first 6 months of life (**D**) and from 6 to 12 months of life (**E**), stratified by infants prenatally exposed (red line) or not (blue line) to malaria. All the Kaplan-Meier survival curves are presented with 95% confidence intervals. *P*-values were determined by log-rank test.

Among infants included in the present analysis, 402 (60.8%) experienced at least one episode of clinical malaria with a median survival time of 9.9 months. Among the covariates from mothers and infants that were assessed for their effect on malaria risk, MiP preventive treatment (Fig. 3A), birth season (Fig. 3B), LBW (Fig. 3C), and PME (Fig. 3D and E) were found to significantly influence malaria risk during the first year of life and, thus, were included as adjusting confounders in the Cox proportional hazard models to assess the association of antibody levels at birth and risk of malaria. Results showed that several antibodies against blood stage antigens, but not against pre-erythrocytic antigens, were associated with malaria protection during the first year of life (Table 3). This was observed for IgG against EBA140 and EBA175 and for cytophilic antibodies (e.g., IgG1 against MSP1_42_ and IgG3 against EBA140 and MSP5). Non-cytophilic antibodies (IgG2 and IgG4) against blood stage antigens were not significantly associated with malaria risk during the first year of life. By contrast, IgG and both cytophilic and non-cytophilic antibodies against markers of PM (i.e., IgG, IgG1, and IgG2 against DBL1-2, and IgG4 against DBL3-4) were associated with an increased risk of malaria episodes during the first year of life (Table 3).

**TABLE 3:** Cox proportional hazards models assessing the association between maternal antibodies in cord blood and the risk of malaria during the first year of life^*a*^ ^,^ ^*d*^

Antigens^*b*^	IgG	IgG1	IgG2	IgG3	IgG4
	HR (95% CI)	HR (95% CI)	HR (95% CI)	HR (95% CI)	HR (95% CI)
Pre-erythrocytic					
CSP-fl	–	–	–	–	–
CSP-Ct	–	–	–	–	–
CSP-NANP	–	–	–	–	–
Erythrocytic					
AMA1	0.54 (0.28–1.04)	–	–	0.88 (0.76–1.02)	–
EBA140	**0.75 (0.60–0.94**)	0.87 (0.75–1.01)	0.85 (0.71–1.03)	**0.85 (0.74–0.97**)	–
EBA175^*c*^	**0.73 (0.57–0.95**)	0.88 (0.77–1.01)	–	–	0.84 (0.70–1.02)
MSP1_42_ ^*c*^	–	**0.70 (0.59–0.85**)	–	–	–
MSP2	–	–	–	–	–
MSP3	–	–	–	–	–
MSP5^*c*^	–	–	–	**0.85 (0.73–0.98**)	–
GARP	–	–	–	–	–
Rh5	–	–	–	–	–
Glycan					
αGal	–	–	0.87 (0.73–1.02)	–	–
Markers of placental malaria					
DBL1-2^*c*^	**1.50 (1.15–1.96**)	**1.27 (1.10–1.46**)	**1.37 (1.09–1.73**)	–	1.20 (0.98–1.46)
DBL3-4^*c*^	–	–	–	–	**1.18 (1.01–1.39**)

^*a*^ HR, hazard ratio; CI, confidence interval.

^*b*^ Only results of Cox proportional models with *P*-value (*P*) ≤0.10 are presented with those statistically significant (*P* <0.05) in bold.

^*c*^ Antigens whose antibody levels were significantly associated with prenatal malaria exposure categories. These models have been adjusted by the variables associated to malaria risk during the first year of life indicated in Fig. 2.

^*d*^ Adjusted hazard ratio and 95% CI for each model are shown.

**Fig 4:**
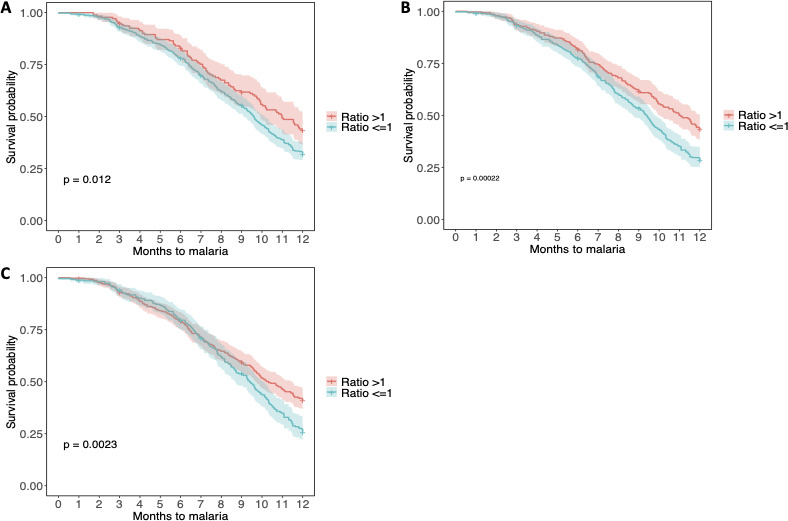
Kaplan-Meier survival curves assessing the effect of ratios between protective antibodies and antibodies associated with increased risk of malaria on infants’ susceptibility to malaria during the first year of life. Kaplan-Meier survival curves (including 95% confidence intervals) are stratified by infants whose ratios were >1 (red line) or equal/below 1 (blue line). (**A)** IgG-EBA140/IgG-DLB1-2 ratio and risk of clinical malaria. (**B)** IgG-EBA175/IgG-DLB1-2 ratio and risk of clinical malaria. (**C)** IgG1-MSP1_42_/IgG1-DLB1-2 ratio and malaria risk. *P*-values were determined by log-rank test.

To further investigate the role of maternal antibodies on malaria susceptibility during the first year of life, ratios between protective antibodies and antibodies associated with increased risk of malaria according to the Cox proportional hazard results were used to assess infants’ risk to clinical malaria (i.e., IgG-EBA140/IgG-DBL1-2, IgG-EBA175/IgG-DBL1-2, and IgG1-MSP1_42_/IgG1-DBL1-2). The proportion of individuals with a ratio >1 and the median ratio were, respectively, 26.6% (176/661) and 3.6 [IQR (1.6–7.9)] for IgG-EBA140/IgG-DBL1-2 ratio, 44.5% (294/661) and 2.4 [IQR (1.4–5.3)] for IgG-EBA175/IgG-DBL1-2 ratio, 59.3% (392/661) and 7.1 [IQR (2.1–34.8)] for IgG1-MSP1_42_/IgG1-DBL1-2 ratio. We found that in all three cases, infants were at a reduced risk of developing a clinical malaria when levels of protective antibodies where higher than those of antibodies associated with an increased risk of malaria (ratios >1) as compared to infants in whom ratios where equal or below 1 (Fig. 4).

## DISCUSSION

In this study, we investigated the relationships between PME, maternal antibody levels at birth, and the risk of malaria during the first year of life using a well-defined birth cohort. Overall, we found that PME had a significant effect on maternal antibody levels in the newborn at birth, and that differences in these antibody levels could drive heterogeneity to malaria susceptibility during the first year of life. Indeed, we observed that PME modified maternal IgG levels in the newborns at different magnitudes, depending on the type of PME, with past PM more frequently associated with significant increase of total IgG and/or subclasses levels across several of the malaria antigens tested. Remarkably, we identified some maternal antibodies associated with differential malaria risk in infancy.

In sub-Saharan Africa, a high number of pregnancies occur annually with a high risk of both peripheral and placental *P. falciparum* infections (56, 57). Of note, in our study population, 75% of children were prenatally exposed to malaria parasites since they were born to mothers with peripheral infection and/or PM. However, there was a very high number of past PM and a very low number of acute PM, probably due to the high use of antimalarial treatments (both, the IPTp-SP, which starts at the beginning of the second trimester, and the infection treatments using AL) as part of the COSMIC trial (34). Differences of MiP strategies between the intervention and the control arms in the COSMIC trial were considered in subsequent analyses. Indeed, children born to mothers in the intervention arm had a reduced risk of experiencing the first clinical malaria episode as compared to those born from mothers in the control arm. It is noteworthy that infants born during the malaria high-transmission season were at higher risk of experiencing a first clinical episode during the first 6 months of life, whereas their counterparts born during malaria low-transmission season had a higher risk from 6 to 12 months of age. Therefore, the high seasonality of malaria transmission in the study area was also taken into account in subsequent analyses.

Antibodies are known to play an important role in host defense against malaria (58). Among these antibodies, total IgG and subclasses are important components of malaria immunity and, in particular, cytophilic IgGs (IgG1 and IgG3) have been more frequently associated with protection from clinical malaria rather than non-cytophilic IgGs (IgG2 and IgG4) (58). However, maternal antibodies thought to protect infants during the first months of life from clinical malaria have been found to be inconsistently associated with protection from field studies (15, 30). Even though it has been reported that babies *in utero* can generate antibodies in response to malaria parasites and soluble antigens crossing the placental barrier, these antibodies represent only a small component of antimalarial antibodies at birth (59, 60). In this study, we identified protective antibodies against four blood stage targets of *P. falciparum* parasites that are key proteins involved in erythrocyte invasion by merozoites: the erythrocyte biding antigens [i.e., EBA140 and EBA175 (61 – 63)] and merozoites surface proteins [i.e., MSP1_42_ and MSP5 (64)], indicating the relevance of maternal antibodies against erythrocytic antigens for infants’ protection against clinical malaria in early childhood. Indeed, higher levels of these antibodies (e.g., IgG to EBA140 and to EBA175, IgG1 to MSP1_42_, IgG3 to EBA140 and to MSP5) were associated with an increased time to first clinical malaria episode during the first year of life. This is in agreement with previous studies that reported association between maternal antibodies against EBAs and MSPs with malaria protection in children (65 – 68), probably by blocking the erythrocyte invasion pathway and/or inhibiting parasites growth (69, 70). Importantly, IgG subclasses against EBAs and MSPs associated with malaria protection were predominantly IgG1 and IgG3 (65, 67, 71).

In contrast, antibodies to DBL antigens (IgG, IgG1 and IgG2 to DBL1-2 and IgG4 to DBL3-4) were significantly associated with an increased risk of clinical malaria during the first year of life. DBL1-2 and DBL3-4 are domains of the VAR2CSA (variant surface antigen 2-chondroitin sulfate A) protein, a variant antigen of the PfEMP1 family that is expressed on infected erythrocytes and mediates parasites sequestration in the placenta through binding to CSA on placental syncytiotrophoblast cells (72, 73). VAR2CSA stands today as the leading candidate for a placental malaria vaccine and DBL1-2 has been assessed in a Phase Ia/Ib clinical trial (53). The presence of antibodies to DBL domains in the cord blood is indicative of PM, and, indeed, antibodies against DBL1-2 and DBL3-4 were increased in PM in this study. PM was also associated with increased risk of malaria during the first year of life, a finding consistent with our previous reports with this same cohort (32, 74) and a number of other studies that showed that PM increases malaria risk during the first months of life (4, 29, 75, 76). Furthermore, this may explain why the active detection and treatment of malaria infection in the COSMIC intervention arm may have reduced the risk of experiencing the first clinical malaria episode as compared to that of the standard IPTp-SP alone among the study participants. Nevertheless, the effect of antibodies to DBL antigens was independent of the effect of PME as models were adjusted by PME.

In spite of the significant increase of antibodies against CSP antigens observed in PM, this study failed to find association between pre-erythrocytic antibodies and malaria protection while recent malaria vaccines studies demonstrated the relevance of these antibodies (in particular the CSP-NANP specific antibodies) in the protection against clinical malaria in children (52, 77, 78). A reason could be that naturally acquired levels of these maternal antibodies to pre-erythrocytic antigens (e.g., CSP-fl, CSP-NANP, and CSP-fl) were not high enough to prevent clinical episodes since their protective effect has been linked to high antibodies titers (79). On the other hand, the fact that antibodies to CSPs are associated with PM (although not as much as for blood stage antigens) is potentially relevant since these maternally transferred antibodies at birth could influence antibody responses to CSP-based malaria vaccines such as R21 and RTS,S through epitope masking, particularly in areas of high malaria transmission intensity (80).

Importantly, several antibodies whose levels were significantly modified by PME were found to be associated with differential malaria risk in infancy (e.g., IgG to EBA175, IgG1 to MSP1_42_, IgG3 to MSP5, IgG4 to DBL3-4, and IgG, IgG1, and IgG2 to DBL1-2). To further assess how maternal antibodies influence malaria susceptibility in these infants exposed to malaria *in utero*, we examined the associations of ratios between protective antibodies and those associated with risk on malaria protection during the first year of life. Based on the Cox proportional hazard analyses results, the effect of two IgG ratios (e.g., IgG-EBA140/IgG-DBL1-2; IgG-EBA175/IgG-DBL1-2) and one IgG1 ratio (e.g., IgG1 to MSP1_42_/IgG1 to DBL1-2) were evaluated, but none for IgG3 since IgG3 levels to both DBL1-2 and DBL3-4 were not significantly associated with malaria risk. Interestingly, we found that despite a prenatal malaria exposure, having higher levels of maternal antibodies against erythrocytic antigens than against DBLs protected children from clinical malaria. Humoral immunity to malaria is complex (58) and these results highlight the multidimensional aspect of the role of maternal antibodies in malaria protection during the first year of life. Further field investigations should include validation of the markers identified here as possible predictors of malaria risk or protection during the first months of life, as well as identification of the combined signatures that are predictive instead of the single antibody approach applied here.

Previous studies have reported a strong correlation between maternal antibodies in the peripheral blood and those in the cord blood at delivery and that these antibody profiles reflect the immunological experience of the mother in her living environment (10, 11, 13). In malaria endemic settings, pregnant women are at high risk of malaria infection that contributes to shape their immunological profile during the course of pregnancy (13, 81, 82). However, pregnancy-associated malaria has different manifestations, probably depending on the time since last infection and the response to it, categorized in four groups in this study (i.e., exposed-no PM, past PM, chronic PM, and acute PM), and shown to be associated to different maternal antibody profiles in cord blood at birth. This different maternal antibody profiles in the newborn may be related to differences in the placental transfer of IgG that is mediated by neonatal Fc receptor (FcRn) expressed on syncytiotrophoblast cells and that depends on (i) maternal levels of total IgG and antigen-specific antibodies, (ii) gestational age, (iii) placental integrity, (iv) IgG subclass, and (v) nature of antigen, being more intense for thymus-dependent ones (83 – 85). Indeed, PME categories have different features which create specific *in utero* environment that probably influences maternal antibodies transfer. Past PM is characterized by persistent hemozoin deposits in fibrin in the placenta with no presence of infected erythrocytes, constituting a biomarker of early infection during pregnancy that has been cleared prior to delivery (86, 87). These early infections during pregnancy may have induced anti-*P*. *falciparum* specific antibodies with sufficient time to cross the placenta, also favored by the resolution of the placenta inflammation and the absence of sequestered parasites, and thus, could explain why past PM was found to be associated with significant increase of IgG and/or subclasses levels in cord blood. In chronic PM, there are infected erythrocytes and hemozoin, and it is interpreted as an infection of longer duration, or a recent placental infection in a pregnant woman with a pre-existing past PM. This could be the reason why chronic PM was associated with an increase of antibody levels against several antigens in bivariable (as illustrated by boxplots in Fig. 2; Fig. S2) but not in multivariable analyses. Also, the lower sample size of this group compared to past PM group might explain to the non-significant association in the multivariable analysis. As expected, these two PME categories showed greater associations with high levels of IgG and subclasses against the markers of PM (i.e., DBL1-2 and DBL3-4) than other categories of PME. On the contrary, it has been reported that PM can decrease transfer of maternal antibodies due to parasite sequestration-related changes of the placenta (88, 89), which could explain why some maternal antibodies against blood stage antigens (e.g., IgG against EBA175 in acute PM, IgG1 against MSP3 in chronic PM and IgG2 against MSP3 and GARP in past PM) were significantly lower in cord blood from mothers with PM than those in the non-exposed control group. Most especially, the serology of the “exposure no PM” group who were born to mothers who experienced malaria during pregnancy but with no evidence of PM by histology at the time of delivery, showed higher levels of IgG to DBL1-2 domains compared to the “non-exposure” group. These findings suggest that this would be an interesting approach to describe malaria exposure during pregnancy for researchers that are working on PM at delivery given that those types of exposure are not detected by histological examination of the placenta.

It’s worth noting that maternal antibody levels decrease over time and, depending on their initial titers, they are expected to be low at 6 months and further decreased at 9 months after birth (5). Thus, the effect of maternal antibodies might be very low from 9 to 12 months. In addition, we previously showed that PME has a profound effect on innate immune system of the newborn and this was associated with malaria risk during the first year of life (32). Therefore, future studies on malaria immunity in early childhood should investigate on maternal antibody decay and the buildup of naturally acquired immunity in children living in high malaria transmission areas with regards to PME categories, as well as the combined effect of fetal innate immunity and maternal antibodies on malaria risk during the first months of life.

In conclusion, these findings indicate that different PME categories have different effects on maternal-derived antibodies levels to malaria antigens in children at birth, and this might drive heterogeneity to clinical malaria susceptibility in early childhood.

## SUPPLEMENTAL MATERIAL

Fig. S1 iai.00268-23-s0001.pdf Spearman correlation matrix between maternal antibodies against the selected antigens.

Fig. S2 iai.00268-23-s0002.pdf Levels of maternal antibodies in cord blood at birth according to PME categories.

Fig. S3 iai.00268-23-s0003.pdf Radar plots showing maternal IgG and subclasses profiles in cord blood by prenatal malaria exposure category.

Table S1 iai.00268-23-s0004.pdf Variables included in linear regression models assessing the effect of PME on maternal antibodies levels in cord blood at birth.

ASM does not own the copyrights to Supplemental Material that may be linked to, or accessed through, an article. The authors have granted ASM a non-exclusive, world-wide license to publish the Supplemental Material files. Please contact the corresponding author directly for reuse.
