# Supplementary material for: Associations between prenatal malaria exposure, maternal antibodies at birth, and malaria susceptibility during the first year of life in Burkina Faso
Source: Infect Immun. 2023 Sep 27;91(10):e00268-23. doi: 10.1128/iai.00268-23 (PMC10580994; doi:10.1128/iai.00268-23)

Supplementary Fig. S1: Spearman correlation matrix between maternal antibodies against the selected antigens.

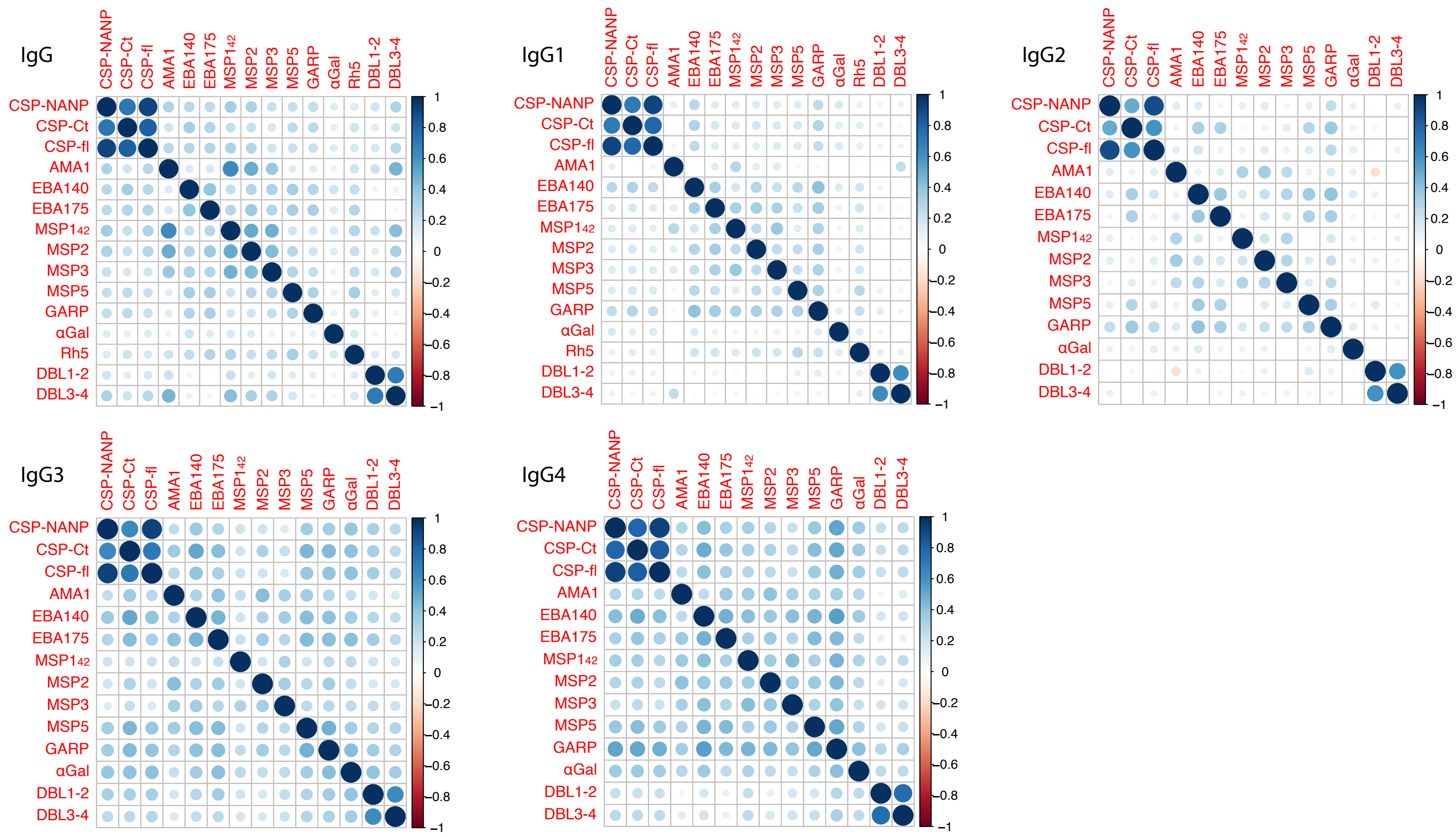

Supplement: Fig. S1 — Spearman correlation matrix between maternal antibodies against the selected antigens. [file iai.00268-23-s0001.pdf]
