# Supplementary material for: Associations between prenatal malaria exposure, maternal antibodies at birth, and malaria susceptibility during the first year of life in Burkina Faso
Source: Infect Immun. 2023 Sep 27;91(10):e00268-23. doi: 10.1128/iai.00268-23 (PMC10580994; doi:10.1128/iai.00268-23)
Supplement: Fig. S2 — Levels of maternal antibodies in cord blood at birth according to PME categories. [file iai.00268-23-s0002.pdf]

Supplementary Fig. S2: Levels of maternal antibodies in cord blood at birth according to PME categories. Boxplots comparing IgG subclasses (IgG1, IgG2, IgG3 and IgG4) levels as $\log_{10}$ of median fluorescence intensity (MFI) between the different PME groups: Non-expo, non-exposed (in green); Expo-no-PM, Exposed/ no placental malaria (in orange); Past-PM, past placental malaria (in blue); Chronic-PM, chronic placental malaria (in pink); Acute-PM, acute placental malaria (in olive green). P-values as determined by Wilcoxon test using the non-exposed group as reference: *≤0.05, **≤0.01, ***≤0.001 and ****≤0.0001. P-values were adjusted for multiple comparisons by Benjamini-Hochberg correction.

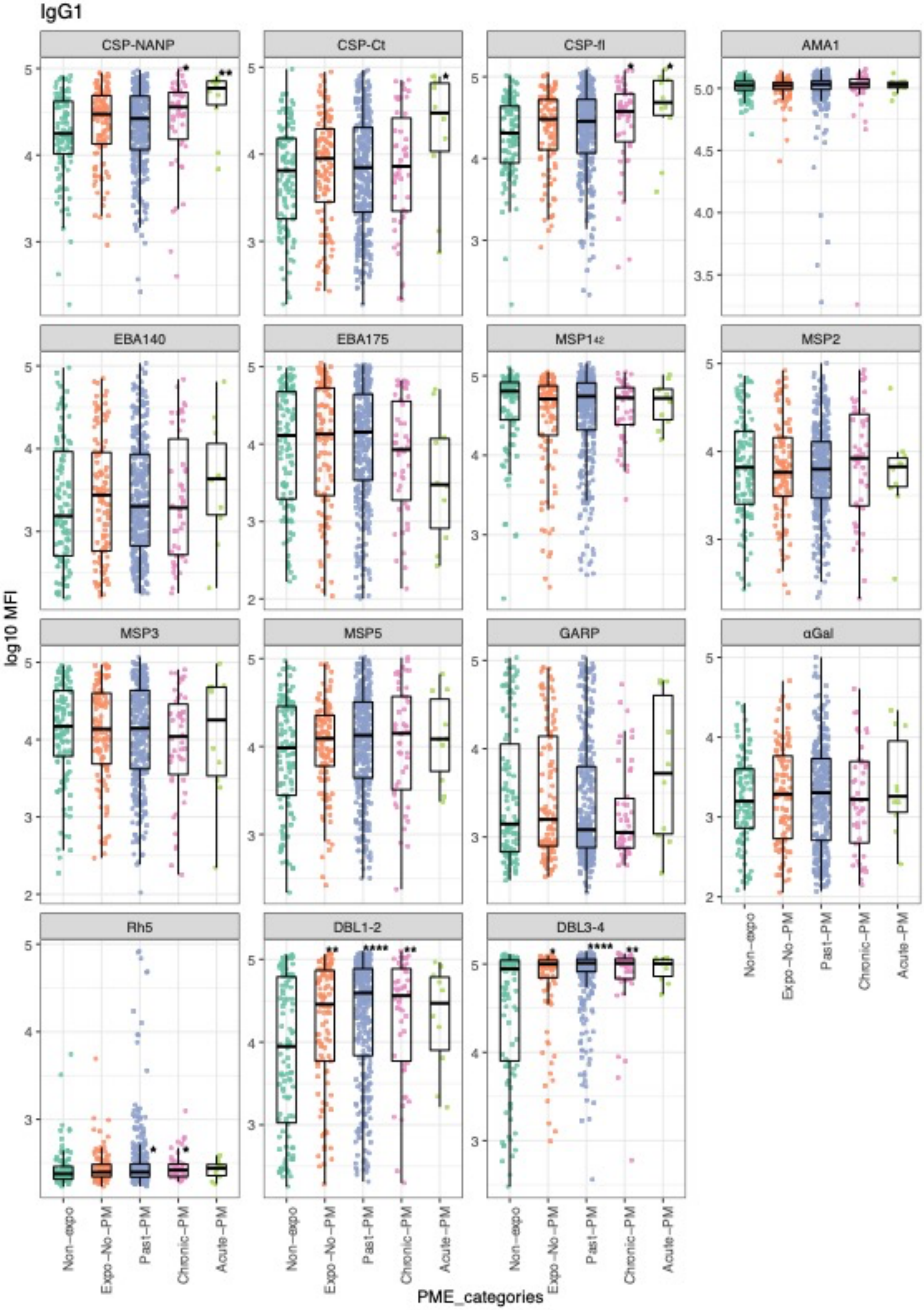

IgG1
CSP-NANP
CSP-Ct
CSP-fl
AMA1
EBA140
EBA175
MSP142
MSP2
MSP3
MSP5
GARP
αGal
Rh5
DBL1-2
DBL3-4
log10 MFI
PME_categories
Non-expo
Expo-No-PM
Past-PM
Chronic-PM
Acute-PM

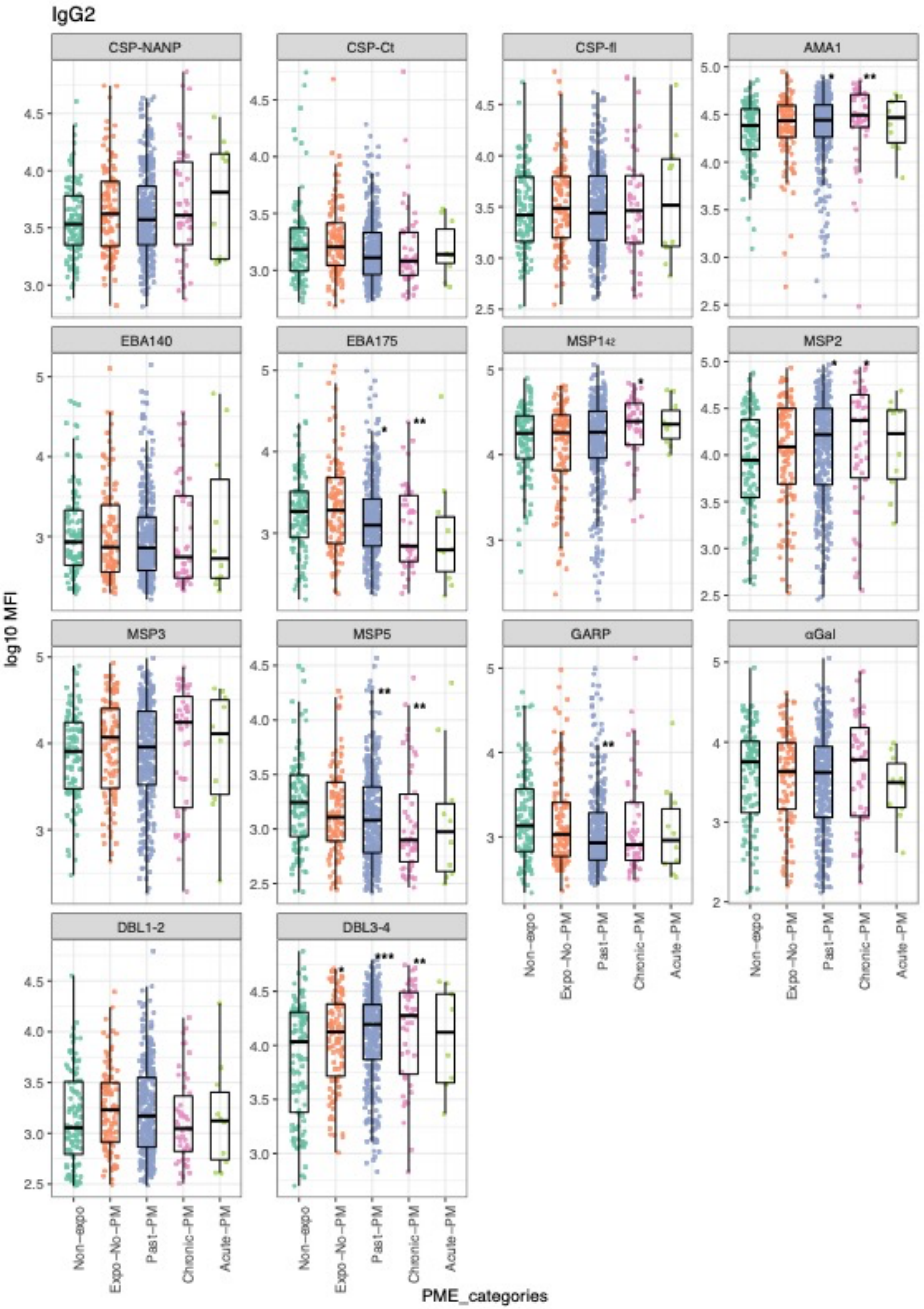

IgG2
CSP-NANP
CSP-Ct
CSP-fl
AMA1
EBA140
EBA175
MSP1 42
MSP2
MSP3
MSP5
GARP
αGal
DBL1-2
DBL3-4
log10 MFI
Non-expo
Expo-No-PM
Past-PM
Chronic-PM
Acute-PM
PME_categories

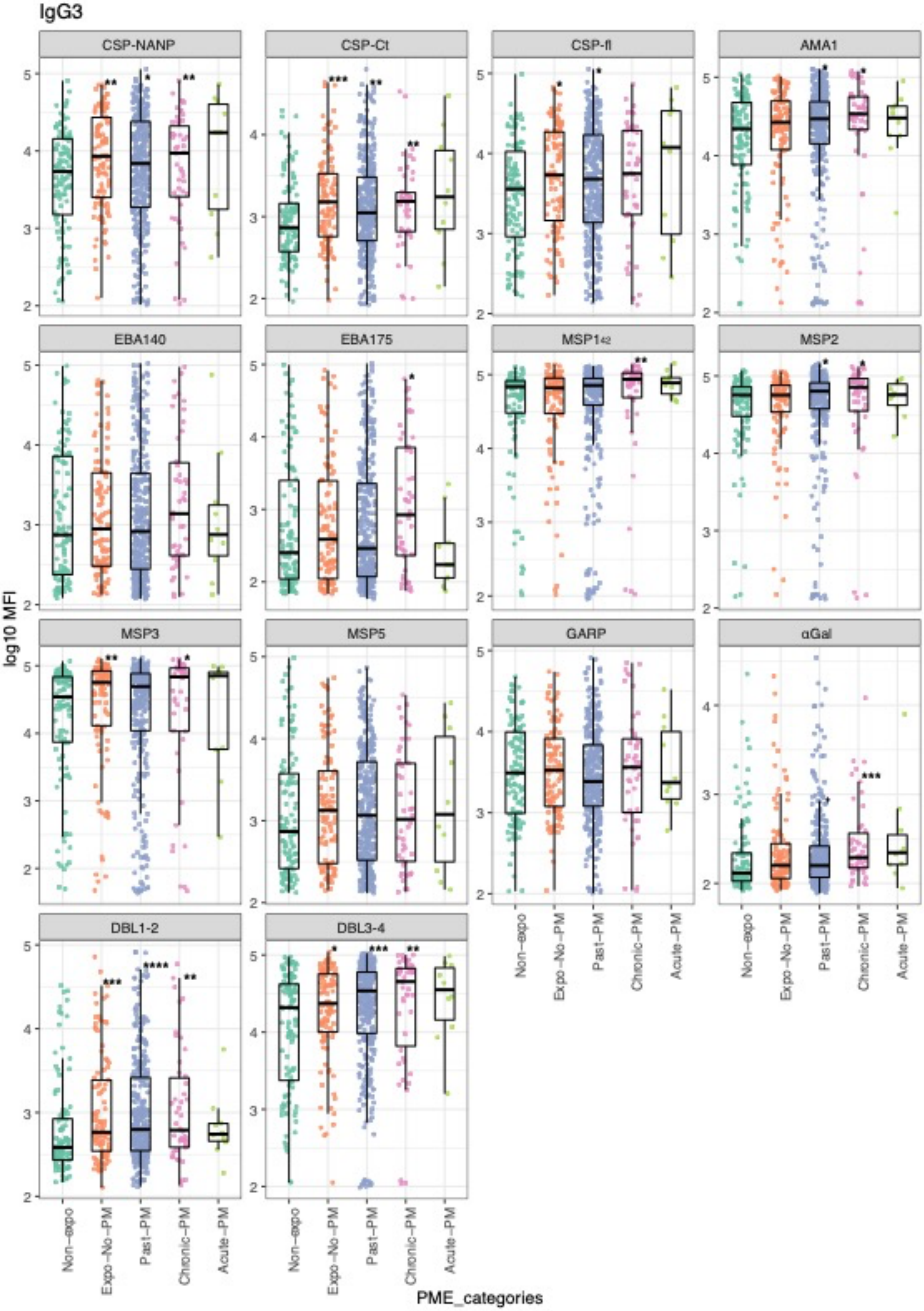

IgG3
CSP-NANP
CSP-Ct
CSP-fl
AMA1
EBA140
EBA175
MSP1₄₂
MSP2
MSP3
MSP5
GARP
αGal
DBL1-2
DBL3-4
log10 MFI
PME_categories
Non-expo
Expo-No-PM
Past-PM
Chronic-PM
Acute-PM

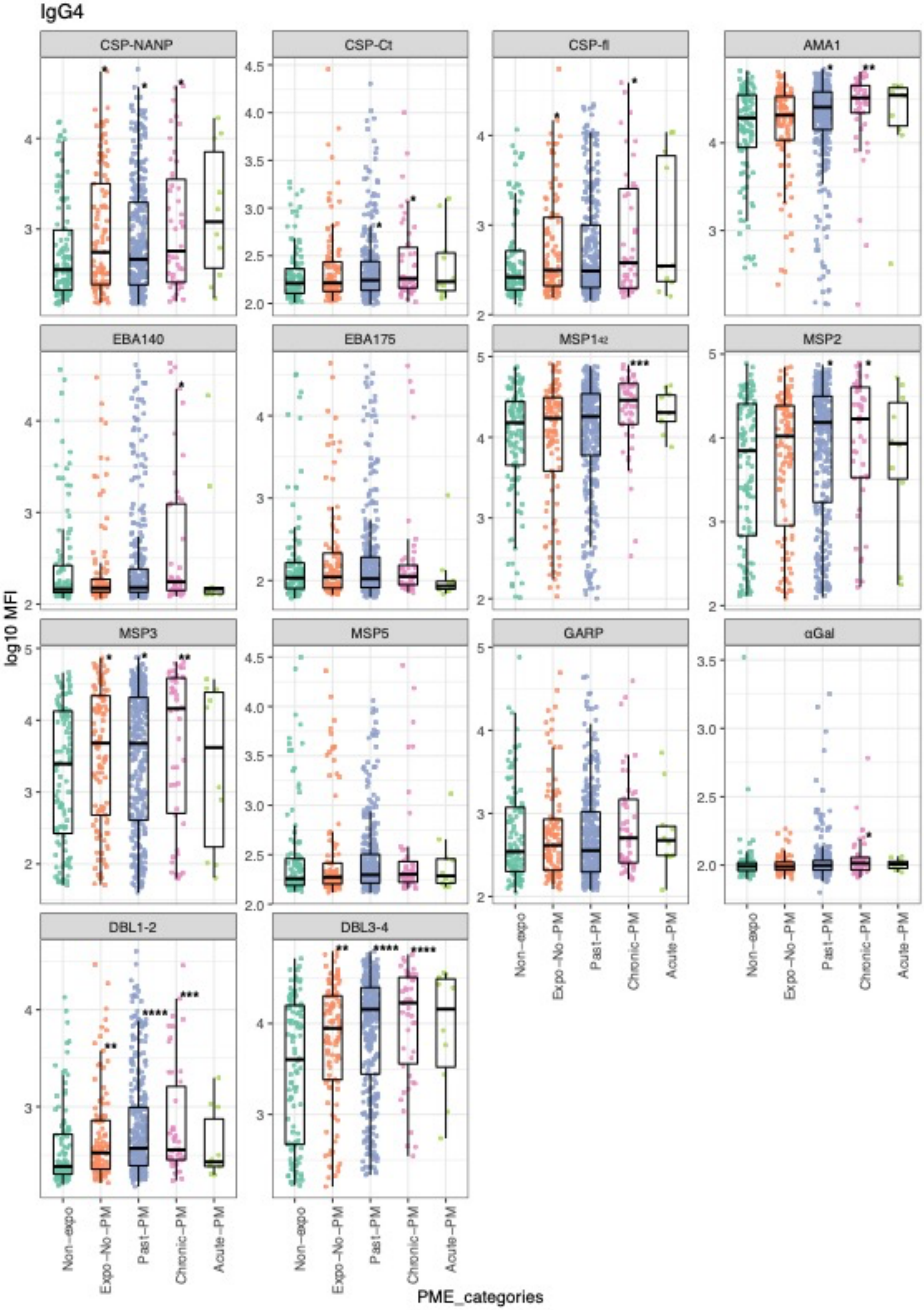

IgG4
CSP-NANP
CSP-Ct
CSP-fl
AMA1
EBA140
EBA175
MSP1₄₂
MSP2
MSP3
MSP5
GARP
αGal
DBL1-2
DBL3-4
log10 MFI
PME_categories
Non-expo
Expo-No-PM
Past-PM
Chronic-PM
Acute-PM
