# Supplementary material for: Associations between prenatal malaria exposure, maternal antibodies at birth, and malaria susceptibility during the first year of life in Burkina Faso
Source: Infect Immun. 2023 Sep 27;91(10):e00268-23. doi: 10.1128/iai.00268-23 (PMC10580994; doi:10.1128/iai.00268-23)
Supplement: Fig. S3 — Radar plots showing maternal IgG and subclasses profiles in cord blood by prenatal malaria exposure category. [file iai.00268-23-s0003.pdf]

Supplementary Fig. S3: Radar plots showing maternal IgG and subclasses profiles in cord blood by prenatal malaria exposure category. Dots represent levels of IgG and subclasses as log_{10} of median fluorescence intensity (MFI) in the different PME groups: Non-expo, non-exposed (in green); Expo-no-PM, Exposed/ no placental malaria (in pink); Past-PM, past placental malaria (in blue); Chronic-PM, chronic placental malaria (in orange); Acute-PM, acute placental malaria (in red).

IgG

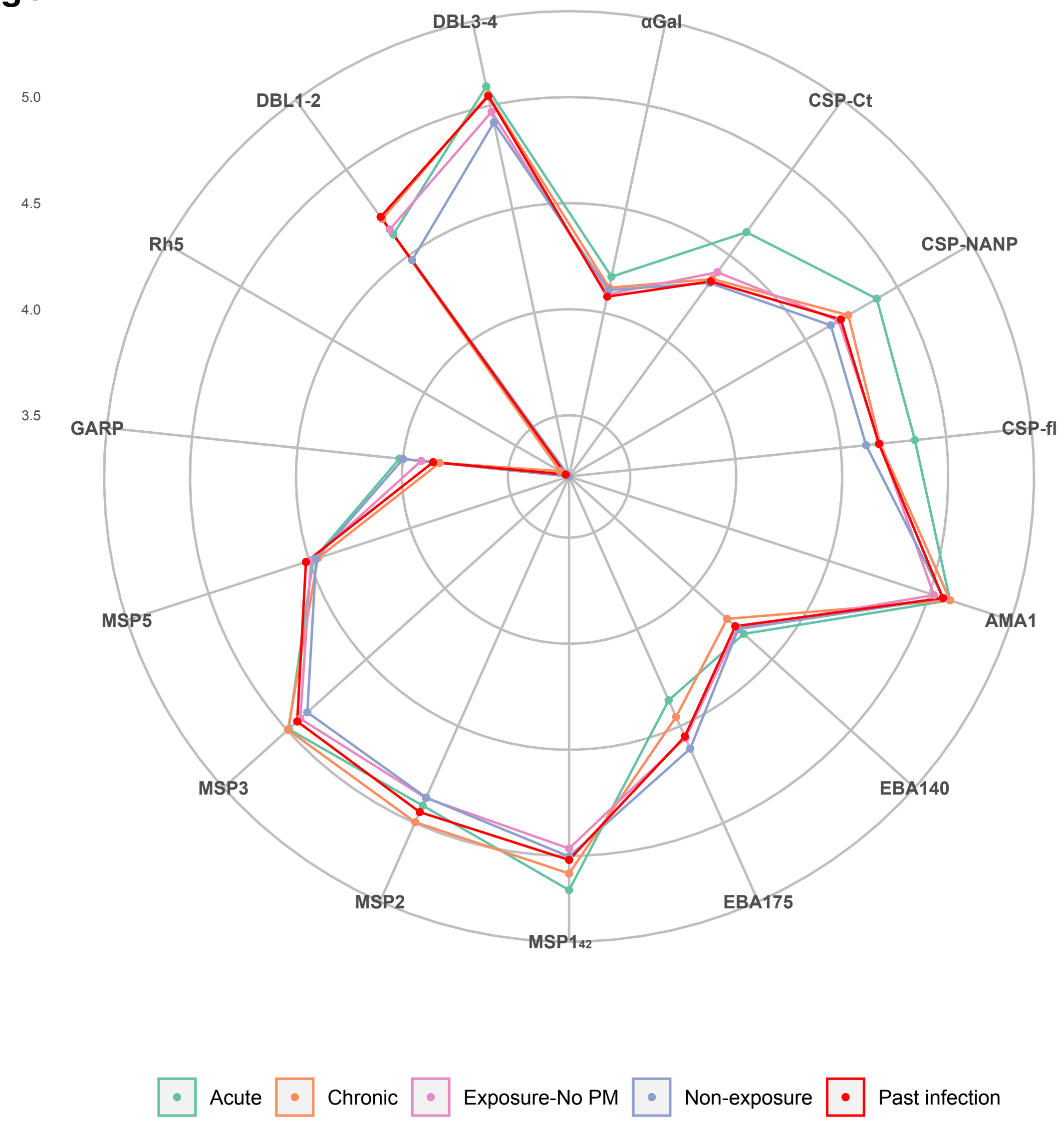

5.0
4.5
4.0
3.5
DBL3-4
αGal
CSP-Ct
CSP-NANP
CSP-fl
AMA1
EBA140
EBA175
MSP1₄₂
MSP2
MSP3
MSP5
GARP
Rh5
DBL1-2
Acute
Chronic
Exposure-No PM
Non-exposure
Past infection

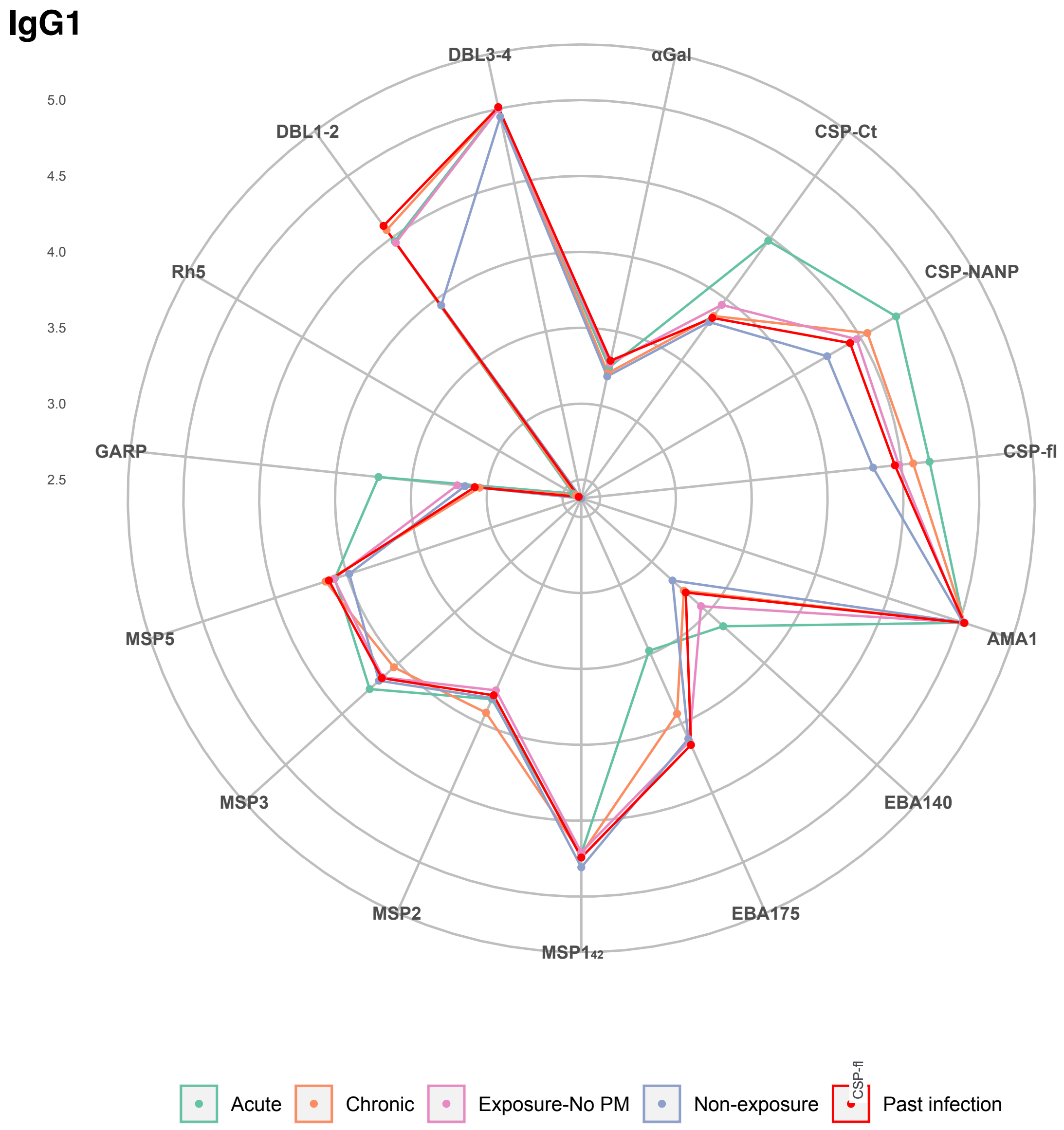
IgG1
5.0
4.5
4.0
3.5
3.0
2.5
DBL3-4
αGal
CSP-Ct
CSP-NANP
CSP-fl
AMA1
EBA140
EBA175
MSP1₄₂
MSP2
MSP3
MSP5
GARP
Rh5
DBL1-2
Acute
Chronic
Exposure-No PM
Non-exposure
Past infection

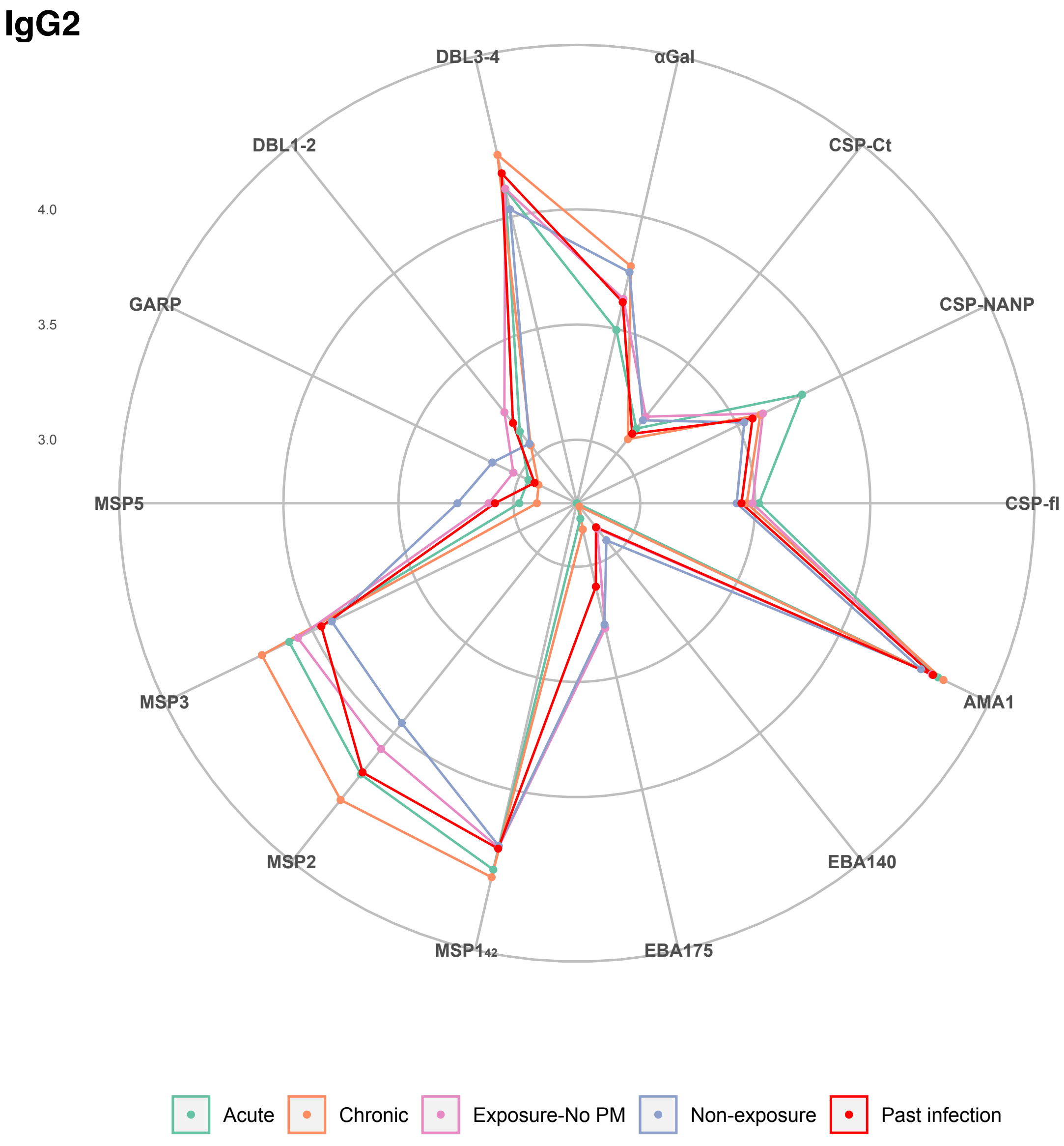

IgG2
DBL3-4
αGal
DBL1-2
CSP-Ct
4.0
GARP
CSP-NANP
3.5
3.0
MSP5
CSP-fl
MSP3
AMA1
MSP2
EBA140
MSP1₄₂
EBA175
Acute
Chronic
Exposure-No PM
Non-exposure
Past infection

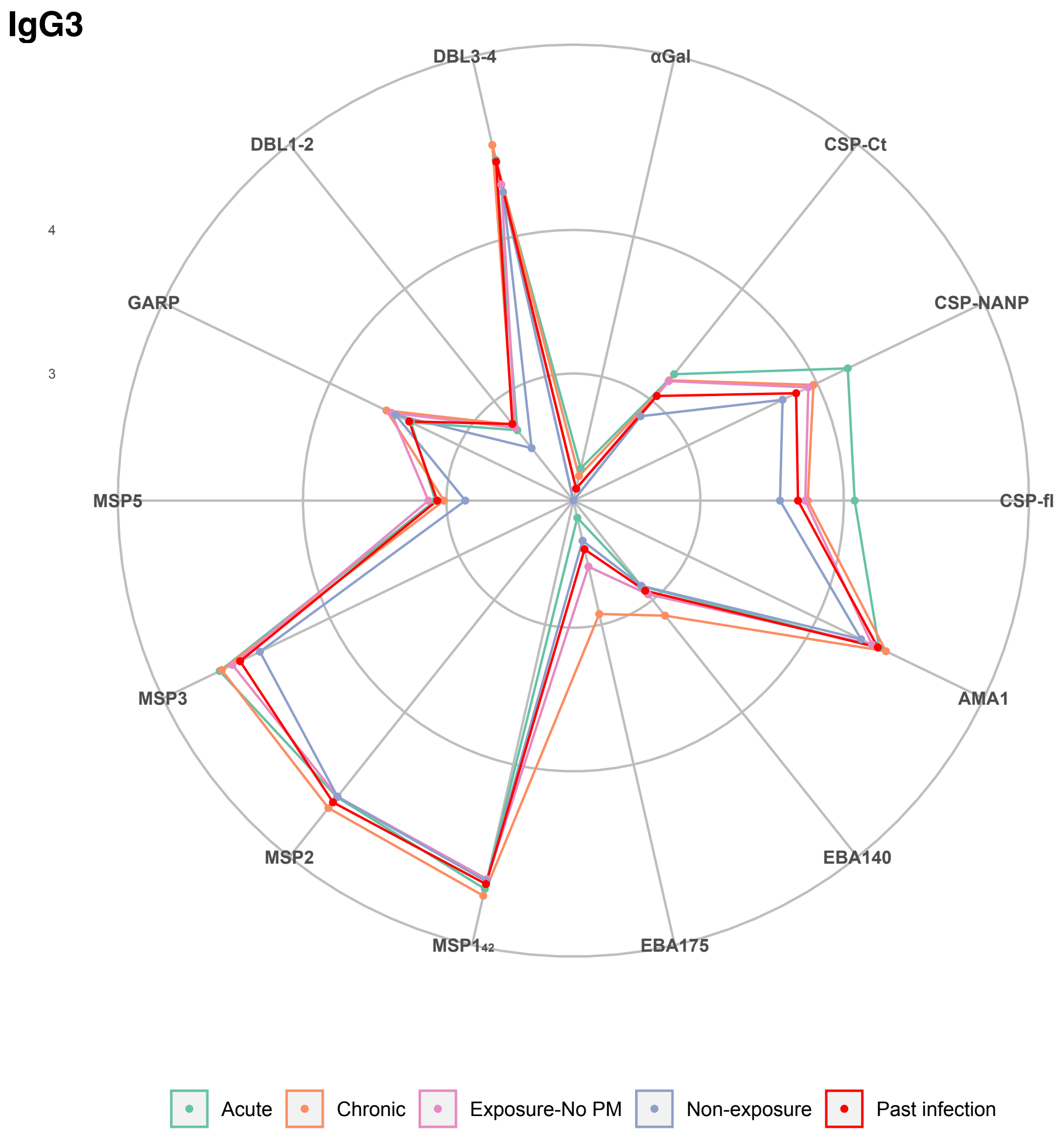
IgG3
DBL3-4
αGal
CSP-Ct
CSP-NANP
CSP-fl
AMA1
EBA140
EBA175
MSP1₄₂
MSP2
MSP3
MSP5
GARP
DBL1-2
4
3
Acute
Chronic
Exposure-No PM
Non-exposure
Past infection

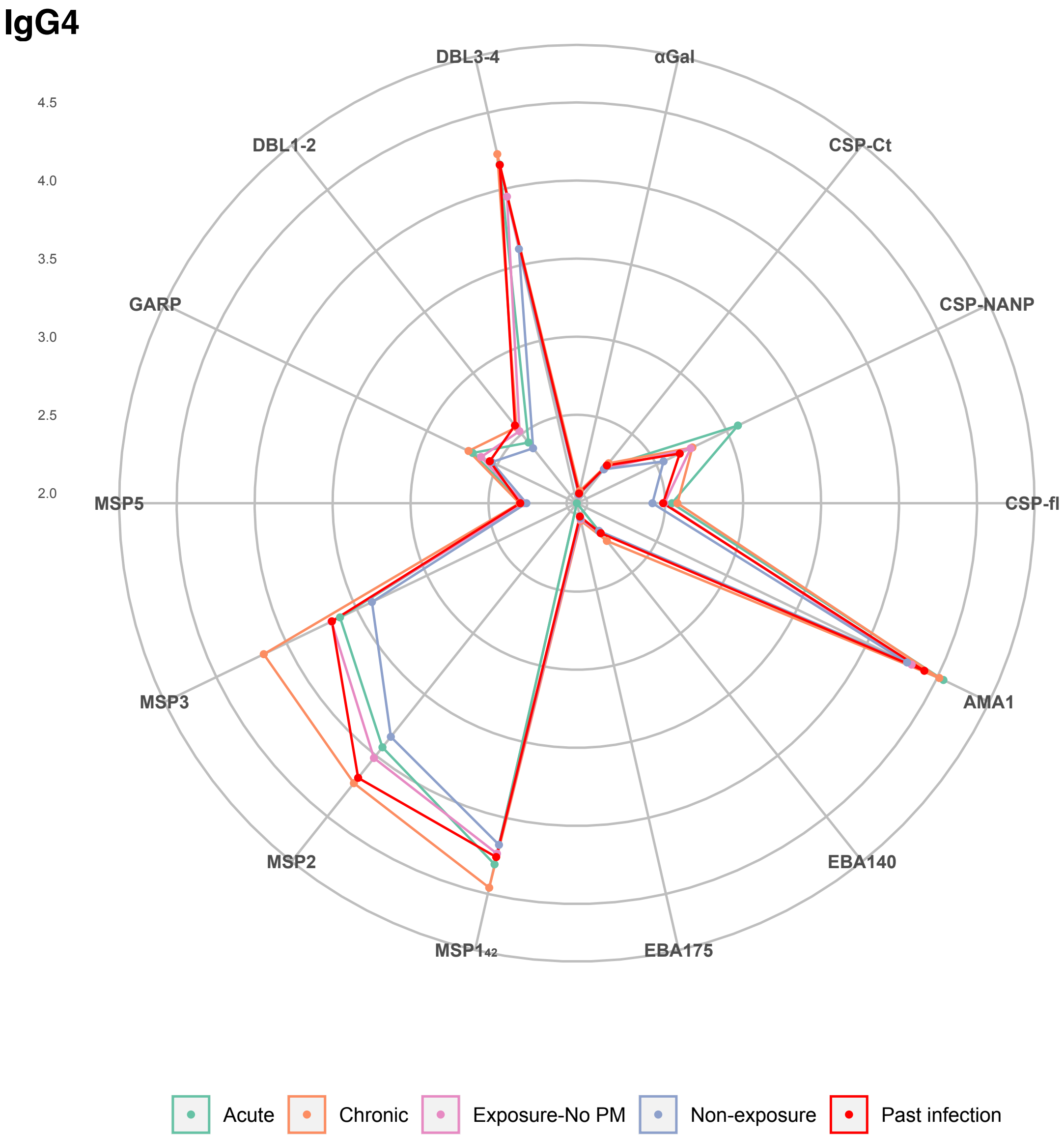
IgG4
4.5
4.0
3.5
3.0
2.5
2.0
DBL3-4
αGal
DBL1-2
CSP-Ct
GARP
CSP-NANP
MSP5
CSP-fl
MSP3
AMA1
MSP2
EBA140
MSP1₄₂
EBA175
Acute
Chronic
Exposure-No PM
Non-exposure
Past infection
