# Supplementary material for: Associations between prenatal malaria exposure, maternal antibodies at birth, and malaria susceptibility during the first year of life in Burkina Faso
Source: Infect Immun. 2023 Sep 27;91(10):e00268-23. doi: 10.1128/iai.00268-23 (PMC10580994; doi:10.1128/iai.00268-23)
Supplement: Table S1 — Variables included in linear regression models assessing the effect of PME on maternal antibodies levels in cord blood at birth. [file iai.00268-23-s0004.pdf]

Supplementary Table S1: Variables included in linear regression models assessing the effect of PME on maternal antibodies levels in cord blood at birth. A backward analysis was used to determine the set of co-factors that significantly associate to antibody levels for each antigen (*i.e.* p value < 0.05 for each co-factor included in the final model).

**Antigens**		**Variables included in the models***				
		**IgG**	**IgG1**	**IgG2**	**IgG3**	**IgG4**
Pre-erythrocytic	CSP-fl	Birth season, gravidity, MiP strategy, ITN use	Birth season, gravidity, MiP strategy, LBW, prematurity, ITN use	Prematurity	**PME, birth season, gravidity, MiP strategy**	Birth season, gravidity, MiP strategy
	CSP-Ct	Birth season, gravidity, MiP strategy, LBW, prematurity, ITN use	**PME, birth season, gravidity, MiP strategy, prematurity**	Birth season	**PME, birth season, MiP strategy**	Birth season, MiP strategy
	CSP-NANP	**PME, gravidity, birth season, MiP strategy, ITN use**	**PME, birth season, gravidity, MiP strategy, prematurity, ITN use**	**PME, Gravidity, Congenital infection**	**PME, birth season, gravidity, MiP strategy**	Birth season, gravidity, MiP strategy
Erythrocytic	AMA1	Birth season	Gravidity, LBW	Gravidity, ITN use	None	Birth season, ITN use
	$MSP1_{42}$	Birth season	**PME, birth season**	None	None	**PME, birth season**
	MSP2	MiP strategy, birth season, ITN use	Gravidity, MiP strategy	**PME, prematurity**	None	Birth season, gravidity
	MSP3	Birth season	**PME, birth season, gravidity**	None	None	Birth season
	MSP5	Birth season	Birth season, gravidity	**PME, birth season, gravidity**	Birth season	Birth season
	EBA140	MiP strategy, ITN use	ITN use	None	MiP strategy, ITN use	Birth season
	EBA175	**PME, LBW, ITN use**	MiP strategy, LBW, ITN use	Birth season, baby sex	**PME, gravidity, MiP strategy**	None
	PfGARP	Birth season, gravidity, Congenital malaria	Gravidity, Congenital malaria, ITN use	**PME**	Gravidity, MiP strategy	Birth season
	Rh5	Birth season	**PME**	-	-	-
Glycan	α-Gal	Birth season	Birth season	None	Birth season	Birth season
Placental Malaria markers	DBL1-2	**PME, gravidity, birth season**	**PME, gravidity**	**PME, birth season, gravidity**	**PME, birth season, gravidity**	**PME, birth season, gravidity, Congenital malaria**
	DBL3-4	**PME, gravidity, birth season**	**PME, gravidity**	**PME, gravidity**	**PME, gravidity**	**PME, birth season, gravidity**

*Covariates of the models include: prenatal malaria exposure (PME), malaria in pregnancy preventive treatment strategy (MiP strategy), insecticide treated net usage by the mother (ITN use), low birth weight (LBW), birth season, gravidity, presence of malaria parasite in the cord blood at delivery (congenital malaria), pre-term birth (prematurity) and ethnicity. Models which include PME are shown in bold.
